# Public Health Questions of To-Day

**Published:** 1905-11-11

**Authors:** 


### The Hospital.

A Journal of

The Medical Sciences and Hospital Administration.

Vol. XXXIX.—No. 998. SATURDAY, NOVEMBER 11, 1905.

### Public Health Questions of To-day.

Among the introductory lectures delivered at the opening of the Medical Session which has just commenced, there was perhaps none more important in itself, and certainly none more important as a source of instruction to the general public, than that which was delivered at the School of University College Hospital by Dr. Kenwood, who combines the office of Professor of Public Health in the College with that of Medical Officer of Health for the Borough of Stoke Newington, and who thus effectively represents both the doctrinal and the practical aspects of the questions with which it is his especial function to deal. Dr. Kenwood strove to impress upon his audience two points of cardinal importance—the first that they could only fully discharge their future duties as practitioners by taking full advantage of the opportunities now afforded them of becoming conversant with hygienic questions; the second that the science and art of sanitation are living and progressive, and that no finality had been attained or is attainable with regard to them. The problems which they offer for solution are changing from day to day, and the devising of practical measures of public health improvement is still hampered in many directions by the imperfection of our knowledge. He pointed out that, owing to paucity of endowments, we have not in Great Britain a sufficient number of workers engaged upon research in sanitary science, and argued that, if there be men who are prepared to devote time, ability, and energy to work of such national importance, those who are favoured with riches should come to their aid in order to make more of such work possible. The view thus stated is one which has been much insisted upon of late in many quarters, and insisted upon, we are disposed to think, without sufficient regard to very strict limitations imposed by nature. In a commercial community any number of persons may become rich, but there is no community in which more than a very few can become capable of conducting research to a successful issue. The intellectual qualifications of a philosopher are not purchasable, and it would be a mistake to allow millionaires to suppose that the successful conduct of research depends upon their liberality. They may honour themselves by smoothing the path of the scientific investigator, as the Emperor Charles V. honoured himself by picking up the brush of Titian, but this is all that they can do. The tone of a good deal of modern writing might lead them to suppose that "research" is something which can be bought and paid for, and the sooner they are disabused of any such impression, the better it will be for all concerned.

Dr. Kenwood deals in a very satisfactory manner with a not uncommon contention to the effect that the practice of hygiene and of preventive medicine tends to the preservation of the physically unfit, and may hence be of but doubtful benefit to the community. He concedes that as a consequence of this practice some weaklings may be permitted to survive, and that, unfortunately, this circumstance affords them increased opportunities for the transmission of their unfitness to future generations. But he maintains that the same conditions which strengthen the weakest make also for the survival of the fittest by increasing the virility of the naturally virile and by tending to maintain the stock in a healthy condition; and that the balance, on the whole, is enormously in favour of sanitation. Preventable disease, moreover, often subtracts from the sum of the vigorous and adds its victims to the sum of the relatively inefficient, for many forms of such disease are indiscriminate in their attack, and often strike down the strongest of the population. If many are now left weak by the passing shadow of disease, and if hygienic measures, although they may raise a few degenerates above the line of viability, at the same time raise the whole of the community in a corresponding degree in the scale of health, it is impossible either to measure the enormous gain or to question the value of the labours of the sanitary reformer.

The endeavours of such reformers, thirty or forty years ago, were chiefly directed to the attainment of structural improvements, to better drainage, better water supply, better ventilation around dwellings, better disposal of refuse. As long as the "housing question" in large towns remains in its present condition it cannot be maintained that the possibilities of useful activity in these directions are exhausted; but Dr. Kenwood thinks that the time has come when the direction of effort should be rather towards persons than towards things. The hygienic reform of the future will, he thinks, depend almost entirely for its success upon the proper education of the people. We are all too painfully aware of the amounts of life and energy which are wasted through ignorance or neglect of the laws of health, and of the consequent misery and loss which are inflicted upon the people. There can be no gainsaying the statement that ignorance of household management and of the elementary principles of hygiene among the poor is in no small measure responsible for their high preventable mortality, their feeble physique, their intemperance, and their poverty. Dr. Kenwood discusses the complex relations which exist between defective education, defective cookery, wasteful and insufficient feeding, and alcoholism; and declares that much of the inability to secure satisfactory food and comfortable homes depends upon the fact that the money which would provide them is spent on drink. Any effective legislation which reduced alcoholism would, he believes, affect beneficially every social public health problem of the day. This is probably true, but it leaves out of sight the pestilent influence of the venal "politician" upon the labouring population, and also the fact that we are nearly as dependent as Russia for our public revenue upon the drinking habits which past legislation has done so much to foster and to promote.

